# Integrated structured light architectures

**DOI:** 10.1038/s41598-020-80502-y

**Published:** 2021-01-12

**Authors:** Randy Lemons, Wei Liu, Josef C. Frisch, Alan Fry, Joseph Robinson, Steve R. Smith, Sergio Carbajo

**Affiliations:** 1 SLAC National Accelerator Laboratory and Stanford University, 2575 Sand Hill Road, Menlo Park, CA 94025 USA; 2 Department of Physics, Colorado School of Mines, Golden, CO 80401 USA

**Keywords:** Adaptive optics, Fibre optics and optical communications, Integrated optics, Fibre lasers, Mode-locked lasers, Ultrafast lasers, Optical manipulation and tweezers, Quantum optics, Ultrafast photonics, Optics and photonics, Frequency combs, Applied physics, Optical physics

## Abstract

The structural versatility of light underpins an outstanding collection of optical phenomena where both geometrical and topological states of light can dictate how matter will respond or display. Light possesses multiple degrees of freedom such as amplitude, and linear, spin angular, and orbital angular momenta, but the ability to adaptively engineer the spatio-temporal distribution of all these characteristics is primarily curtailed by technologies used to impose any desired structure to light. We demonstrate a laser architecture based on coherent beam combination offering integrated spatio-temporal field control and programmability, thereby presenting unique opportunities for generating light by design to exploit its topology.

## Introduction

Structured photonics lay the foundation for the generation or use of light with custom spatio-temporal variant field vector, amplitude, and phase distribution. Coloration in peacock feathers and photonic band structures in butterfly wings are among the many complex morphologies of light commonly found in nature. In recent decades, artificial structuring of light has undergone a remarkable evolution to produce orbital and spin angular momenta beams exhibiting unique properties such as optical vortices and topological vector fields^1, 2^. Beyond areas of recent decadal impact, such as optical communications^3^, sensing^4^, and particle trapping^5^, today these properties are examined to create transformational tools in molecular physics^6–8^, quantum^9^, relativistic^10, 11^, and nonlinear optics^12^, and particle physics^13^, to name a few. Unconventional ways of conceptualizing light structure are inspiring new families of electromagnetic fields that circumvent generally unquestioned functional properties. For example, Bessel-Bessel-Bessel light bullets^14^ can propagate without appreciable diffraction distortion, while an ensemble of finite-energy wavepackets is capable of abruptly focusing and defocusing outside the paradigm of paraxial optics^15^. As quanta, spatio-temporally variant topological states of light, particularly if they can be changed dynamically, could enable photonic and information technologies in quantum computation and highly-selective light-matter interactions, including Floquet insulators^16^, photonic skyrmions^17^ or two-dimensional metasurface polaritons^18^.

While the outlook for applications of structured photonics is promising, their fruition is hampered by bottleneck technologies that unable to fully exploit all degrees of freedom to advance the generation of light with adaptable structure. One common way of engineering structured light is by using spatial light modulators. These devices can control the intensity and phase of a light beam in an image or Fourier space. They represent the success story of light shaping technologies and their widespread application in holographic display technology and optical tweezers, for example. Some key parameters that also define light structure are often inaccessible using light shaping technologies, such as temporal intensity distribution of light bullets or pistons, or active control of the carrier-envelope phase. But perhaps their main limitation is their operational damage threshold, especially unfavorable for ultrashort pulse manipulation, thereby impeding progress on structured light applications where moderate to extremely high peak- or average-power levels are at play, above MW- and W-levels, respectively^19^, including strong-field laser-matter interactions, directed energy, and free-space optical communications among others.

Coherent combination of femtosecond pulses is a realistic power-scalable configuration with kW-level powers demonstrated to date^20^. Phased arrays have been studied as an alternative source and successfully demonstrated coherent synthesis of beams with varying degrees of freedom^21–25^. In particular, vortex^23, 25^ and orbital angular momentum (OAM)^21, 22^ beams have been demonstrated employing a variety of circularly symmetric arrays in combination with phase masks. These demonstrations are based on phase modulation between beams only, excluding continuous amplitude modulation and active polarization and carrier-envelope phase (CEP) modulation, which are capable of producing a larger family of synthesized beams. This motivates re-examining the ability to produce more generalized structured light bullets, in addition to vortex and OAM beams, using tiled phased arrays by incorporating all parameters defining the 3-dimensional wavevector distribution, not only phase and amplitude but also CEP, pulse front, and polarization, in a programmable or adaptable fashion.

**Figure 1:**
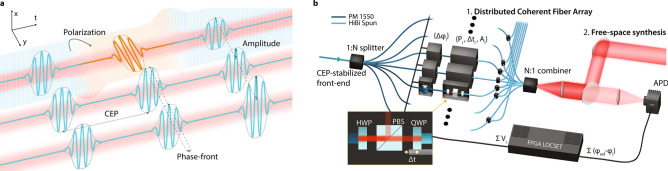
(**a**) conceptual depiction of a transversely and longitudinally coherent optical comb and the corresponding primary elements that define how the electromagnetic field distribution will synthesize; and (**b**) the experimental configuration via coherent multi-channel coherent fiber array with a common CEP-stabilized front end, independent phase ($\Delta \phi_{i}$), amplitude ($A_{i}$), polarization state ($\wp_{i}$), and timing ($\Delta t_{i}$) controls, and active locking via FPGA LOCSET using a single avalanche photodiode (APD) in the far-field. The output coherent output can be delivered in the form of a distributed coherent fiber array or the form of a free-space synthesized pulse.

In this letter, we present a generalized laser architecture and experimental demonstration that enables the design of light bullets with built-in programmable structure to be exploited adaptively. This architecture capitalizes on the synthesis of phased arrays with individually controllable field-amplitude, carrier-envelope and relative phase, and –polarization. We refer to each of these elements of the array as beamlines. The underlying principle of this configuration is illustrated in Fig. 1.a, where the primary field properties can be controlled in a spatio-temporal optical comb. When a coherent relationship is maintained between all the beamlines, these combs can be made to collapse and generate unique spatio-temporal wavevector distributions.

The proof-of-concept consists of ${\text{N}}$ = 7 + 1 ($i = 1:{\text{N}}$) fiber-based beamlines, each split from a femtosecond mode-locked laser operating at the C-band telecom wavelength range (Fig. 1*.*b). The front-end is CEP stabilized using an ultralow phase-noise feed-forward technique^26^ that ensures single-digit mrad pulse-to-pulse jitter^27^ for practically long operation^28^. Stabilizing the CEP is the first step to guarantee pulse-train absolute phase consistency across all beamlines. After splitting the CEP-stabilized front-end, one beamline sets a reference in order to monitor and control the relative inter-beamline phase offset via a self-synchronous and self-referenced custom field-programmable gate array (FPGA) phase-locking technique. This configuration enables all 7 beamlines to be phase-locked to the absolute reference phase—which is set via CEP—by any arbitrary phase relationship. Thus, all but the reference beamline undergo active manipulation and monitoring of all the measurable field parameters—namely phase (${\Delta }\phi_{i}$), amplitude (${\text{A}}_{i}$), polarization state ($\wp_{i}$), and timing (${\Delta }t_{i}$)—prior to their coherent synthesis or distributed delivery. Each beamline contains a phase modulator (here a piezoelectric transducer-based fiber stretcher) that imposes a user-defined phase relationship with respect to the reference beamline via computer-interfaced FPGA with a maximum programmable range of 20 in the case of these specific modulators. Active phase-locking provides 40 mrad phase noise, or 33 as of timing jitter, corresponding to an outstanding level of stability four orders of magnitude lower than the entire programmable range. The intensity and polarization vector control units for each beamline consist of a half waveplate, polarizing beam splitter, and quarter waveplate placed on a fiber pigtailed delay stage for timing. After individual manipulation of the field vectors, circularly birefringent fibers preserve each beamline’s final polarization state prior to synthesis. The composite beam is collimated and synthesized in free space with a micro-lens array in a tiled-aperture configuration with the seven beamlines arranged hexagonally to be spatio-temporally overlapped at a photodiode. This photodiode is the only optical detection component required for the self-synchronous self-referenced locking technique. Further technical details can be found on Methods. The resulting product is a laser architecture that can deliver programmable laser pulses in the form of a free-space synthesized light bullet (e.g. near or far-field), as an array of distributed coherent beamlines (e.g. fiber), or as hybrid distributed fiber- and free-space beamlines.

**Figure 2:**
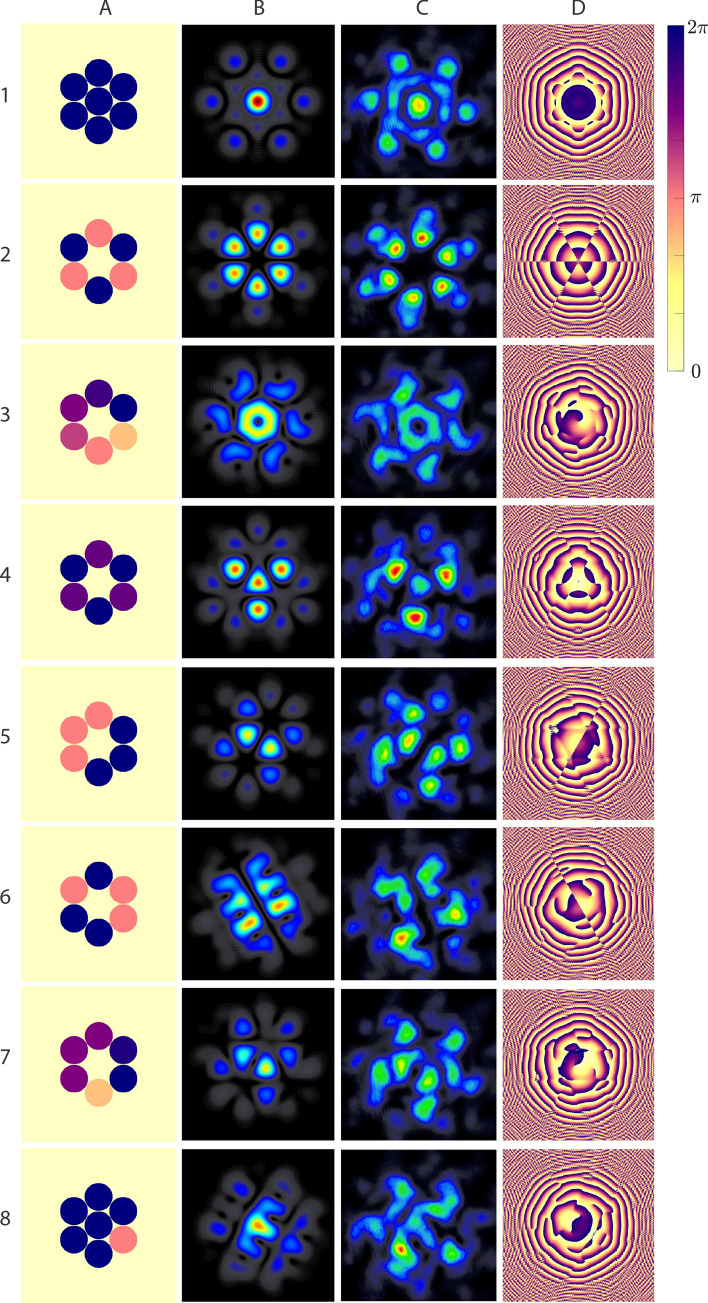
Near-field phase- and amplitude combinations (column **A**) and their corresponding retrieved (column **B**) and measured synthesized far-field intensity (column **C**) and phase distributions (column **D**).

**Figure 3:**
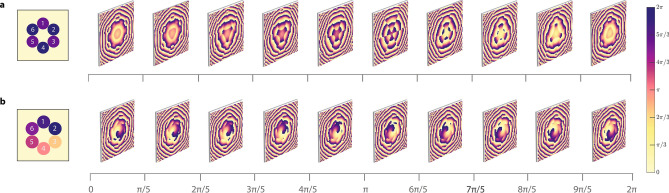
Evolution of the phase-front for two near-field case scenarios where (**a**) the CEP is locked and the relative phase of channels $k$ = 1,3,5 is varied from 0 to $2\pi ;$ and (**b**) the relative phase of all channels $k$ = 1,...,6 is locked with $\phi_{k} = 2\pi \left( {k - 1} \right)/6$ and the CEP is varied from 0 to $2\pi$.

Figure 2 exemplifies the synthesis of various pulses in the far-field from combining only the amplitude and relative phase of the beamlines. The near-field hexagonal arrangement is chosen here for demonstration purposes only. In order to highlight the effectiveness of these two knobs alone in generating complex intensity- and phase- distributions, we denote each beamline’s relative phase difference with respect to the others ($\phi_{k}$) to be $\phi_{k} \le 2\pi$ and for simplicity, the amplitude of each beamline ($A_{k}$) to be either ‘on’ or ‘off’, i.e. $A_{k} \in \left\{ {0,1} \right\}$, where $k \in Z\left[ {1,N} \right]$ and $N$ is the total number of beamlines in the combined output. The various $A_{k}$ and $\phi_{k}$ arrangements are displayed on column A in Fig. 2. Columns B and C show the corresponding calculated and measured far-field transverse intensity distributions, respectively. Column D is the corresponding retrieved phase distribution at the plane of the measurement. Row 1 exemplifies a conventional coherently combined beam, typically used for intensity scaling purposes. Rows 2–4 demonstrate cylindrically structured pulse synthesis with either alternating or gradually varying phases. For instance, in Row 3 the phase is defined as $\phi_{k} = 2\pi \left( {k - 1} \right)/N$, that is, the total phase offset spans over $2\pi$ increasing clock-wise monotonically and uniformly, which is equivalent to generating a discretized first-order orbital angular momentum (OAM) beam. Note that while both the far-field intensity and phase resemble that which is expected, i.e. a Laguerre–Gauss and helical shapes, respectively, the additional structure in these distributions arises naturally from discretization. As the distributions in Row 2 and 3 are brought to the far field, a phase singularity appears at the center of these beams. It is worth noting that this singularity shifts away from the center towards the three triangular-equidistant lobes in the case of Row 4. The direction of increasing phase in the near field can be switched to change the chirality of the OAM beam in the far field. Despite the hexagonal arrangement, cylindrically asymmetric pulses can also be generated, as shown in Rows 5–8, some of which result in abrupt phase transitions with reflectional symmetry. Collectively, these examples underscore the ability to create adaptive and dynamic field singularities and intensity distributions. For applications requiring higher finesse than demonstrated with a 7-channel configuration in free-space synthesis, we compare the modeled results in Fig. 2, Row 3 for a higher and feasible number of channels in Methods, Fig. 3.

**Figure 4:**
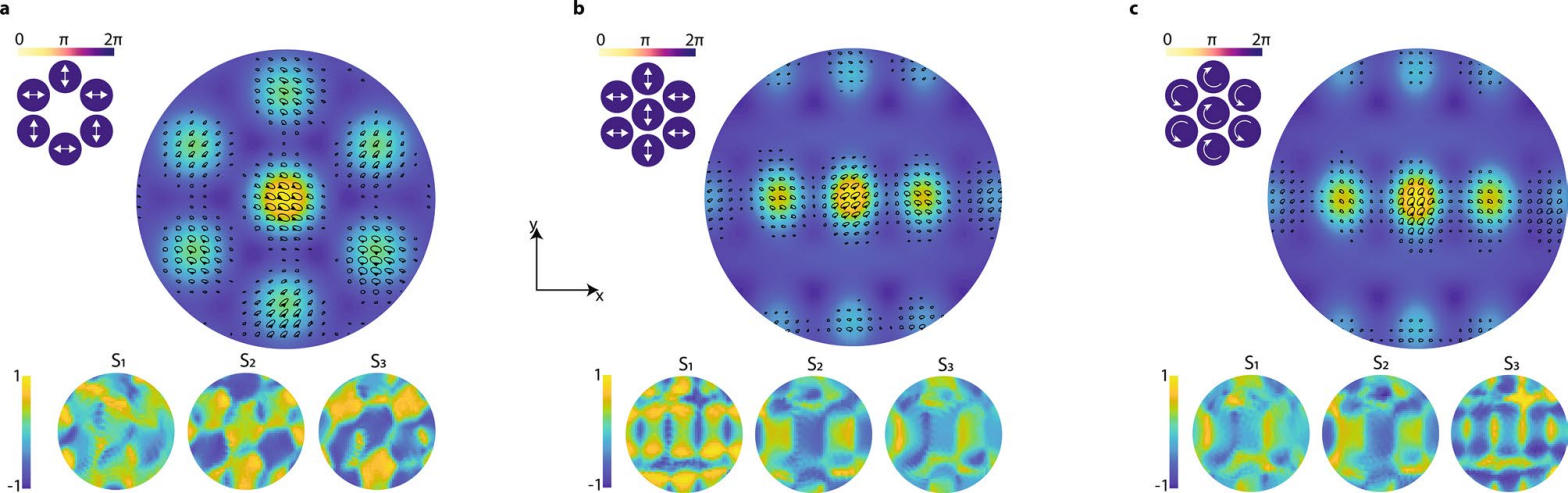
Experimental results of polarization topography evolution with corresponding near-field configurations (top left) and Stokes projections (bottom) for alternating linear (**a**), asymmetric linear (**b**), and asymmetric circular polarization coherent synthesis (**c**).

In Fig. 4, we showcase the adaptive evolution of the phase-fronts enabled by both CEP and relative LOCSET phase control. We simulate the far-field phase distribution of two near-field arrangements shown in Row 2 and 3 of Fig. 2. In the first case, shown in Fig. 4a and corresponding to Row 2 (Fig. 2), we vary the relative phase of channels $k =$ 1,3, 5 from 0 to $2\pi$ simultaneously, while keeping the other three channels fixed at the same phase. In practice, the resulting phase-front evolution corresponds to gradually shifting the relative phase of channels $k =$ 1,3, 5 without modifying the CEP, which sets the absolute phase value for the remaining channels. As another example, if the LOCSET is preset to impose a cylindrical near-field phase distribution (as in Fig. 2 Row 3) and only the CEP is shifted over a single optical cycle, shown in Fig. 4b, then a helical phase-front rotating along the propagation axis is synthesized. In Figs. 2 and 4 examples, only first-order topological charges can be reliably generated due to the low (7 + 1) discretization employed. To reproduce higher-order OAM beams or complex vector vortices, a higher number of channels will likely be required according to end-user needs, which can be examined employing our beam propagation model (see Methods). The representation of these phase-front evolutions alongside their corresponding near-field and far-field intensity distributions may be better visualized in the animations found in Supplementary Information.

**Figure 5:**
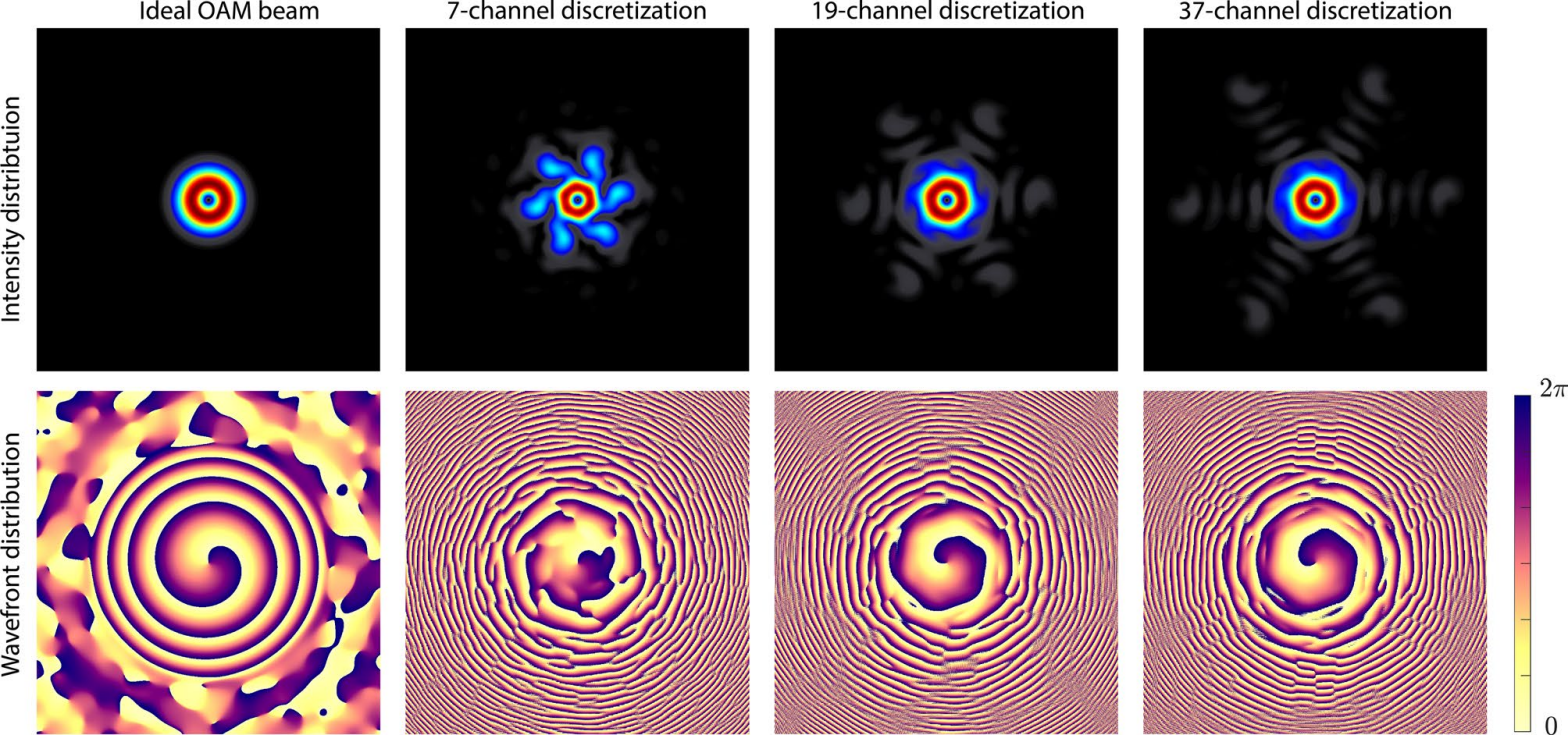
Modeled first-order OAM beam intensity (first row) and wave front (second row) distributions for the ideal representation (first column) and 7-, 19-, and 35-channel hexagonal configurations (second through fourth columns, respectively).

Another salient feature of the architecture is the capacity to generate programmable composite phase-fronts with various array polarization states, thereby producing beams with spatially and temporally variant spin angular momentum distributions, also known as polarization topography. We show the evolution of a few topographic maps in Fig. 5a–c. Each of these cases illustrates a 2D projection of the spin angular momentum distribution. The distribution is overlaid on top of the far-field intensity distribution with the corresponding near-field configuration (top left) alongside the three measured Stokes’ maps on the bottom. In these examples, we have chosen to maintain all beamlines set to approximately the same phase and amplitude value and focus on the non-uniform transverse polarization topography generated solely by varying the near-field polarization distribution. In these examples, the topographic maps evolve to follow constructive interference and a degree of ellipticity and chirality determined by the collective contribution of all beamlines at any transverse (x,y) point in the 2D projection. Combining beamlines with orthogonal polarization states facilitates the generation of stable and adaptable interference patterns with alternating topological charge and singularity regions in the transition from one charge to another. The measured Stokes projections display the transferability of one spin distribution to another, confirmed by the inverse relationship between the linear polarization projection map in (b) and the circular projection map in (c), that is, ${\text{S}}_{1}^{{\text{b}}} = - {\text{S}}_{3}^{{\text{c}}}$. Because the transverse polarization distribution arises from any combination of ${\Delta }\phi_{i}$, ${\text{A}}_{i}$, and $\wp_{i}$, an exceptionally large ensemble of topographic maps is possible with only a few channels. More dynamical examples of the topographic polarization can be found in Supplementary Information.

One last consequential feature of this integrated structure light architecture resides entirely on the composite action of all ${\Delta }t_{i}$, which facilitate the generation of optical pistons from small delays (few to several wavelengths) with a time precision determined by the locking phase stability to very large delays beyond the duration of the pulses and dynamic range determined by optical delay stages. It is important to note that while all examples presented above are synthesized in the free space to highlight and diagnose the main architectural capabilities, channel delivery need not only collapse in a single location but can also be configured as a distributed coherent array in any guided, unguided, or hybrid configuration. In the case of free-space synthesized beams, we have also developed a numerical method for the reconstruction and optimization of complex field synthesis^29, 30^ beyond the presented examples, which is particularly important for non-conventional and unintuitive forms of structured light. This modeling tool can assist any end-user in reconstructing the optimal system configuration that renders any desired user-defined synthesized beam.

This demonstration materializes a dynamical and programmable architecture to produce light by design, where spatio-temporal wavevector distributions can be tailored in real time to enable further exploration of structured photonics and its applications. In particular, we demonstrate the synthesis of high geometrical dimensionalities of structure, including nontrivial vector map topologies in spatial and temporal dimensions. The system is self-encapsulated such that field controls (${\Delta }\phi_{i}$, ${\text{A}}_{i}$, $\wp_{i}$,${{ \Delta }}t_{i}$) are integrated in the architecture itself. It also presents a few notable imminent opportunities such as power-scalability and hyperspectral extension via integrated photonics to be examined in quantum electrodynamics and on-chip accelerator drivers^31^. More broadly, this architecture aspires to seed new frontiers of light control and manipulation, optical quantum communications and information processing, as well as emerging concepts in nonlinear topological and nuclear photonics.

## Methods

### Carrier-envelope phase-stabilized front-end and beamline controls

We use a soliton mode-locked Er:Yb:glass laser oscillator (OneFive Origami-15) and CEP stabilization based on a feed-forward (FF) system^27^. The oscillator delivers 140 mW of power in 175 fs pulses at a repetition rate of 204 MHz ($f_{REP}$) with a spectral bandwidth of 14.9 nm centered around 1.55 µm. The light from the oscillator is split into two beamlines: one towards the in-loop (IL) feedback measurement and the other through the acoustic-optic frequency shifter (AOFS) and towards the out-of-loop (OOL) measurement. Both beamlines are coupled into stretcher fiber, which is spliced with Er:fiber amplifiers. After nonlinear amplification, the pulse is recompressed and passes through highly nonlinear fiber (HNLF) for octave spanning. The spectrally broadened pulses are coupled out to free space and frequency-doubled in a periodically-poled lithium niobite tuned for second harmonic generation at 1024 nm. The light is then passed through optical bandpass filters centered at 1024 nm and focused onto an avalanche photodiode (APD).

The raw signal from the IL APD is sent to the FF electronics and conditioned for the AOFS, which has an operational frequency of 80 ± 2.5 MHz, since $f_{CEO}$ is not guaranteed to be in this range. The signal is filtered to isolate $f_{CEO}$ with 40 dB SNR (RBW:100 kHz), which is mixed with a local oscillator (LO) and amplified to 26 dBm. The final signal is given by $f_{AOFS} = f_{CEO} + f_{LO} = 80 MHz$. The AOFS subtracts the drive signal from the frequency comb replacing $f_{CEO}$ with $f_{LO}$ and power is shifted to the AOFS -1st diffraction order and coupled into the OOL interferometer fiber. The raw signal measured in the OOL interferometer contains $f_{LO}$, $f_{REP}$, and mixing products. For jitter analysis, the signal is filtered to remove $f_{REP}$ and mixing products.

Long term operation is achieved by sending to $f_{AOFS}$ to a slow feedback loop that adjusts the pump power of the oscillator to correct for drifts in the system. The loop detects drift away from 80 MHz through mixing with a stable 79.8 MHz signal, a low pass filter at 500 kHz, and a frequency to voltage (F2V) converter. The F2V signal is then fed to a PID controller with only proportional and integral gains. The output of this PID is finally what drives the pump power changes in the oscillator.

We employ SPUN-HiBi fibers for individual channel delivery. These fibers are designed to preserve circular polarization. The composite beam is collimated with a microlens array in a tiled-aperture configuration arranged hexagonally. Microlens arrays can be fabricated with sub-micrometer precision, and thus readily enable reproducible alignment in free-space synthesis configuration.

Relative time overlap/delay is achieved using a fiber pigtailed delay stage in each channel. The intensity is modulated by a half waveplate and a polarizing beam splitter integrated in the delay line. Phase control is achieved by means of 7 phase modulators (PZT-based fiber stretcher) capable of operating at bandwidths larger than 10 kHz.

### Multi-channel phase modulation: FPGA-based LOCSET

Optical phase control and modulation begin with the alignment of the incoming optical phase of the seven channels, accomplished by overlapping all channels on a single photodiode (PD) and maximizing the amplitude seen by the diode. An optical phase error signal for each channel is generated by phase modulating (PM) each channel at a unique PM frequency of a few hundred Hz to tens of kHz. Therefore, the LOCSET technique is an appropriate choice for channel and power scalability because it can accommodate active control and phase-locking in excess of hundreds of channels^32^. One beam may serve as a phase reference without modulation, though convergence is slightly faster if the feedback is applied to all channels driving them all to the mean phase of the ensemble (referenceless operation). Front-end electronics down-convert the PD electrical signal from 204 MHz, corresponding to the nominal repetition rate of the laser, to 2 MHz and digitized at 20 MSPS by a 16-bit ADC whose output is streamed to the feedback FPGA. The FPGA digitally demodulates the 2 MHz IF then further demodulates each sideband at the PM frequencies of each of the seven channels. These sidebands may be coherently demodulated as the FPGA itself synthesizes the PM drive. The sign and amplitude of each sideband give the optical phase error for each channel. Each beamlet phase error drives a feedback loop filter for that beamlet. The feedback bandwidth may be set as high as a few hundreds of Hz, so phase convergence is attained quickly. Once optical coherence has been established at the photodiode, one can program the desired complex optical modulation program. As optical path lengths drift, optical phases must periodically realign by recohering the beams on the photodiode.

### Beam propagation model

The free-space simulation of this system is a discrete fast Fourier transform angular spectrum evaluation method of the Rayleigh-Sommerfeld diffraction formula. The object plane for all simulations is placed at the micro-lens array and assumes that all beamlines are collimated. Additionally, we place the image plane at our camera and assume that the propagation between the two planes satisfies all requirements for using a scalar rather than vector propagation theory^33^. The use of angular frequencies, and the resultant finite, discrete grid, introduces conditions on the proper sampling of the object and image planes, and their reciprocal spaces to obtain accurate results. First, all non-zero values of the objects must be included in the computational grid which is automatically satisfied if the extent of the grid is larger than the object^34^. Second, the maximum propagation distance without aliasing due to under sampling is related to the real space grid spacing, the wavelength of light, and the farthest extent in the grid which contains a non-zero value^35^. Third, the minimum propagation distance must be substantially greater than the wavelength^33^. Since our propagation is on the order of meters the third condition is satisfied and we simply increase the extent of the object grid to a large enough size that the second condition is met. Additionally, we model the full image plane of mixed polarization states at the object plane by propagating two fields, one for each orthogonal polarization, and taking the sum of their intensity distributions at the image plane.

The numerical model for the reconstruction and optimization of complex field synthesis is publicly accessible and can be found in the following references^29, 30^.

### Polarization vector map calculations

The polarization vector maps are reconstructions of the local polarization ellipse generated from stokes parameters. In order to capture the Stokes parameters $\left\{ {{\varvec{S}}_{0} ,{\varvec{S}}_{1} ,{\varvec{S}}_{2} ,{\varvec{S}}_{3} } \right\}$, seven images, the full field and one for each of the six projections on the Poincaré sphere, are mixed according to the known definitions. Each projection image of size ${\mathbb{N}} \times {\mathbb{M}}$ is captured by system consisting of a quarter-wave plate, a half-wave plate, a polarizing beam splitter, and an InGaAs camera. To ensure that each projection image is capturing the same region of the field a smaller image of ${\varvec{n}} \times {\varvec{m}}$ pixels is taken from the full image and the normalized cross-correlation between this and each projection is calculated. When the cross-correlation is at its maximum the projection is assumed to be capturing the same region and then cropped to ${\varvec{N}} \times {\varvec{M}}$. Next to remove errors from shot to shot pixel differences the images are subdivided into ${\varvec{n}} \times {\varvec{m}}$ macro-pixels, where each macro-pixel contains the mean of a subset of true pixels ${\varvec{\alpha}} \times {\varvec{\beta}}$ such that $\user2{\alpha n} = {\varvec{N}}$ and $\user2{\beta m} = {\varvec{M}}$. After being centered and sub-divided, the seven images are finally used to calculate the local Stokes parameters of the field. To generate the polarization ellipse, it is necessary to know the eccentricity, the tilt relative to a fixed axis, and the chirality. The eccentricity is given by,

1 $${\varvec{e}} = \sqrt {\frac{{2\sqrt {{\varvec{S}}_{1}^{2} + {\varvec{S}}_{2}^{2} } }}{{1 + \sqrt {{\varvec{S}}_{1}^{2} + {\varvec{S}}_{2}^{2} } }}}$$

the tilt by,

2 $$2{\varvec{\theta}} = \tan^{ - 1} \frac{{{\varvec{S}}_{2} }}{{{\varvec{S}}_{1} }}$$

and the chirality is determined from the sign of ${\varvec{S}}_{3}$. In Fig. 5, we reduced the number of plotted vectors to reduce visual clutter without eliminating the shape of the evolving field. More generally, complex spatial distributions of orbital angular momentum and polarization can be quantified and represented by mapping the phase and Poynting vectors, as described by Ref ^36^.

### The role of channel discretization in free-space synthesis

This section compares and quantifies the effect of discretization, i.e. number of channels in the free-space synthesis configuration. While not end-user applications require the same amount of finesse and beam quality, it is important to demonstrate this effect using one of the examples in Fig. 2, a vortex beam of first order.

We employ the same reconstruction and optimization model described above^29, 30^ to model the intensity and wavefront distribution of an OAM beam of first order in its ideal form, 7-channel (as demonstrated) configuration as well as 19- and 37-channel configurations. The last two configurations are chosen based on the number of channels required for the next two additional layers in a hexagonal configuration such that the far-field distributions can be compared to one another.

Visibly, the diffractive contributions outside of the ring-shaped intensity distribution are reduced with increasing the number of discretization, thereby resembling a higher topological charge purity. The high-frequency structure in the wavefront also decreases with increasing number of channels to approximate a spiral distribution, as expected. We also calculate the mean squared error (MSE) of the intensity distributions between the ideal and each discretized beam as a fitness metric. The results show that the MSE between the ideal and 7-channel beams is 0.0016, while this value is reduced to 0.001 and 0.0006 for the 19- and 37-channel configurations, respectively.

## Supplementary information

Supplementary Information 1.

Supplementary Information 2.

Supplementary Information 3.

Supplementary Information 4.
