# Supplementary material for: Integrated structured light architectures
Source: Sci Rep. 2021 Jan 12;11:796. doi: 10.1038/s41598-020-80502-y (PMC7804322; doi:10.1038/s41598-020-80502-y)
Supplement: Supplementary file 4 — Supplementary Information 4. [file 41598_2020_80502_MOESM4_ESM.docx]

Integrated Structured Light Architectures

Randy Lemons^1,2^, Wei Liu^1^, Josef C. Frisch^1^, Alan Fry^1^, Joseph Robinson^1^, Steve R. Smith^1^, Sergio Carbajo^1,2§^

^1^SLAC National Accelerator Laboratory and Stanford University, 2575 Sand Hill Road, Menlo Park, CA 94025, USA

^2^Department of Physics, Colorado School of Mines, Golden, CO 80401, USA

§corresponding author: [scarbajo@stanford.edu](mailto:scarbajo@stanford.edu)

Movie 1.mp4: First dynamical example of the topographic polarization with alternating linear polarizations in a near-field hexagonal configuration. In this configuration, out of 6 channels in a hexagon with half are vertically polarized and the other half horizontally polarized. The phase offset of the vertically polarized channels is ranged from 0 to 2π

Movie 2.mp4: Second dynamical example of the topographic polarization with first-order OAM beam with a spiral wavefront and rotating near-field linear polarizations. The near-field is a rectangular array with a Laguerre-style rotation pattern such that rotation angle for each channel starts at 0 in the bottom right corner and goes in steps of π/4 moving clockwise. Only the center channel is turned and its rotation angle changes from 0 to π.

Movie 3.mp4: Third dynamical example of the topographic polarization with using a 2x3 rectangular array. All channels have a rotation angle of π/4; left-side channels have an increasing phase offset while the right-side channels have a decreasing phase offset from 0 to π.
